# Evaluating the Impact of the HeartHab App on Motivation, Physical Activity, Quality of Life, and Risk Factors of Coronary Artery Disease Patients: Multidisciplinary Crossover Study

**DOI:** 10.2196/10874

**Published:** 2019-04-04

**Authors:** Supraja Sankaran, Paul Dendale, Karin Coninx

**Affiliations:** 1 Expertise Center for Digital Media Hasselt University Diepenbeek Belgium; 2 Department of Cardiology Heart Center Jessa Hospital Hasselt Belgium; 3 Faculty of Medicine and Life Sciences Hasselt University Diepenbeek Belgium

**Keywords:** heart diseases, cardiac rehabilitation, human factors engineering, evaluation studies, telerehabilitation, mobile app, multidisciplinary research

## Abstract

**Background:**

Telerehabilitation approaches have been successful in supporting coronary artery disease (CAD) patients to rehabilitate at home after hospital-based rehabilitation. However, on completing a telerehabilitation program, the effects are not sustained beyond the intervention period because of the lack of lifestyle adaptations. Furthermore, decline in patients’ motivation lead to recurrence of disease and increased rehospitalization rates. We developed HeartHab, using persuasive design principles and personalization, to enable sustenance of rehabilitation effects beyond the intervention period. HeartHab promotes patients’ understanding, motivates them to reach personalized rehabilitation goals, and helps to maintain positive lifestyle adaptations during telerehabilitation.

**Objective:**

This study aimed to investigate the impact of the HeartHab app on patients’ overall motivation, increasing physical activities, reaching exercise targets, quality of life, and modifiable risk factors in patients with CAD during telerehabilitation. The study also investigated carryover effects to determine the maintenance of effects after the conclusion of the intervention.

**Methods:**

A total of 32 CAD patients were randomized on a 1:1 ratio to telerehabilitation or usual care. We conducted a 4-month crossover study with a crossover point at 2 months using a mixed-methods approach for evaluation. We collected qualitative data on users’ motivation, user experience, and quality of life using questionnaires, semistructured interviews and context-based sentiment analysis. Quantitative data on health parameters, exercise capacity, and risk factors were gathered from blood tests and ergo-spirometry tests. Data procured during the app usage phase were compared against baseline values to assess the impact of the app on parameters such as motivation, physical activity, quality of life, and risk factors. Carryover effects were used to gather insights on the maintenance of effects.

**Results:**

The qualitative data showed that 75% (21/28) of patients found the HeartHab app motivating and felt encouraged to achieve their rehabilitation targets. 84% (21/25) of patients either reached or exceeded their prescribed physical activity targets. We found positive significant effects on glycated hemoglobin (*P*=.01; *d*=1.03; 95% CI 0.24-1.82) with a mean decrease of 1.5 mg/dL and high-density lipoprotein (HDL) cholesterol (*P*=.04; *d*=0.78; 95% CI 0.02-1.55) with a mean increase of 0.61 mg/dL after patients used the HeartHab app. We observed significant carryover effects on weight, HDL cholesterol, and maximal oxygen consumption (VO_2_ max), indicating the maintenance of effects.

**Conclusions:**

Persuasive design techniques integrated in HeartHab and tailoring of exercise targets were effective in motivating patients to reach their telerehabilitation targets. This study demonstrated significant effects on glucose and HDL cholesterol and positive carryover effects on weight, HDL cholesterol, and VO_2_ max. There was also a perceived improvement in quality of life. A longer-term evaluation with more patients could possibly reveal effectiveness on other risk factors and maintenance of the positive health behavior change.

**Trial Registration:**

ClinicalTrials.gov NCT03102671; https://clinicaltrials.gov/ct2/show/NCT03102671 (Archived by WebCite at http://www.webcitation.org/76gzI9Pvd)

## Introduction

### Cardiac Telerehabilitation

A global increase in multiple risk factors such as inactive lifestyles, obesity, diabetes, hypertension, high cholesterol, and smoking is contributing to the higher occurrence of coronary artery disease (CAD). Secondary prevention through cardiac rehabilitation (CR) programs has proven to be effective in reducing these risk factors and thereby reducing the risk of recurrence of disease and rehospitalization [1-3]. Despite these established benefits, the uptake of and adherence to these programs remain low. Distance to rehabilitation centers, time, cost, and psychological barriers are some of the main reasons for reduced uptake of such rehabilitation programs [4,5]. Telerehabilitation includes the use of technology-based approaches to monitor patients remotely [6], alongside facilitating the delivery of other rehabilitation components [7], while they rehabilitate independently after their hospital-based rehabilitation phase. Telerehabilitation approaches enable us to overcome issues in conventional rehabilitation such as distance to rehabilitation center, being restricted to fixed time and appointments, and cost per session [8,9]. However, in both conventional and telerehabilitation approaches, it has been demonstrated that effects of rehabilitation programs are not sustained after stopping the intervention [3], although the rate of decline in effects is lower in telerehabilitation when compared with conventional rehabilitation [10]. In addition, patients do not maintain positive lifestyle adaptations enforced during rehabilitation after the end of the program, leading to increased risk of recurrence.

### Previous Work

Multiple studies have proven the benefits of telerehabilitation approaches from different perspectives. Reviews and studies have shown that technology-supported CR approaches are as effective as conventional hospital-based rehabilitation approaches [11-13]. Some studies also prove the cost-effectiveness of engaging in such interventions [9,14]. However, as highlighted earlier, the rate of decline of effects upon completion of intervention is lower in telerehabilitation as compared with conventional rehabilitation [14]. Psychological studies have shown that effectively implementing behavior change interventions supports habit formation and facilitates in sustaining motivation over a longer period [15-17]. Furthermore, when it comes to technology-supported interventions, it is important to design systems that are user-friendly and accessible. To enhance user experience by considering user (patient) needs and perspectives, human-computer interaction (HCI) studies mainly focus on the usability and interaction techniques when designing technology-supported rehabilitation systems. The consideration of patient-specific needs and perspectives can facilitate in tailoring the delivery of these technology-based interventions to cater precisely to the target patients, thereby minimizing attrition.

### Goal of the Crossover Study

This study evaluates the effectiveness of an app-based multidisciplinary telerehabilitation program on 4 core factors associated with CR: F1) patients’ perspectives, experience, and impact on motivation; F2) impact on physical activity; F3) impact on quality of life; and F4) impact on risk factors.

## Methods

### App Design

We developed HeartHab, a comprehensive patient-tailored app to support cardiac telerehabilitation. HeartHab covers various modules related to CR such as monitoring risk factors (blood pressure, weight, glucose, cholesterol etc), medication management, physical activity training, e-coaching via specially designed videos, and symptoms monitoring. The design of HeartHab’s user interface is grounded on persuasive design techniques drawn from both psychology and HCI literature. The key principle of designing persuasive systems is drawn from the Fogg Behavior Model [18], Persuasive Systems Design model [19], and the Behavior Wizard [20]. On the design level, persuasive design patterns, which are suitable in the context of CR, were derived from the above theories and integrated into visual elements presented to patients such as their risk factor thresholds, activity targets and progress, and so on [21]. On an application level, persuasion is achieved through intelligibility, personalization, and tailoring of various rehabilitation components. The app remotely connects to a caregiver’s dashboard used by cardiologists, nurses, and physiotherapists to remotely monitor patients’ risk factors physical activity progress, prescribe medication, tailor physical activity targets, and receive timely alerts and notifications of each patient. A video walkthrough of various components of HeartHab is presented in Multimedia Appendix 1.

In our previous work, we studied the usability of HeartHab and patient perceptions of the persuasive techniques used in multiple field studies [10,22,23]. Having validated the usability of the app and having gained promising insights on the influence of intelligibility, tailoring, and other persuasive design elements toward motivating patients, we wanted to assess the actual impact on the aforementioned CR factors (F1-F4). For this, we used a multidisciplinary crossover approach by combining clinical and HCI evaluation methods. By following such an approach, we go beyond conventional randomized controlled trials and usability studies to do a mixed-methods evaluation and present both qualitative and quantitative outcomes. Furthermore, the crossover approach facilitates in getting detailed insights in a shorter period with considerably lower number of participants. We did not allow for a wash-out period in this specific context as one of our key objectives was to study if there were any crossover effects at the end of an intervention to observe maintenance of effects after the termination of the intervention. The crossover impact also presents indications of maintenance and longer-term outcomes. In this paper, we describe the approach and results of the evaluation in detail including a detailed statistical analysis of crossover effects that were specifically observed.

The medical ethical committees of Hasselt University and Jessa Hospital approved the study. The patients signed an informed consent form before startup and were allowed to withdraw from the study at any point.

### Recruitment

**Figure 1:**
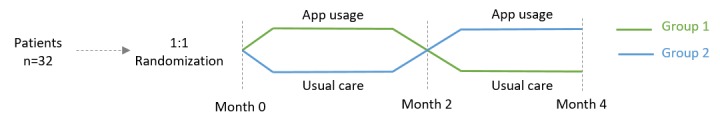
The process of randomization showing change in phases of the study for both groups before and after crossover.

A total of 50 cardiac patients who met the inclusion criteria (Textboxes 1 and 2) were screened on the cardiology database of a regional hospital to participate in the study. They were informed of the study details and, if agreeable to participate in the study, asked to sign the consent form. Overall, 32 patients consented to participate in the study and were randomly assigned in a 1:1 ratio to 1 of the 2 treatment strategies (usual care or using the HeartHab app; Figure 1) between April and June 2017. Patients randomized to group 1 used HeartHab in the first phase and received usual care in the second phase, whereas patients randomized to group 2 received usual care in the first phase and HeartHab in the second phase. Each phase of the study was for a period of 2 months (Figure 1). In the usual care phase, patients did not receive any form of rehabilitation support and continued the usual self-management without any supervision.

**Inclusion criteria for patient recruitment in the study.**

**Inclusion criteria:**

- History of coronary artery disease with or without intervention (percutaneous coronary intervention or coronary artery bypass grafting or conservative)
- History of a cardiac rehabilitation program
- Clinically stable without inducible ischemia or high-risk ventricular arrhythmia, confirmed by the last available maximal ergo-spirometry test
- Aged over 18 years
- Willing and physically able to follow an app-based telerehabilitation program and other study procedures in a 4 months follow-up period
- Possession of and/or able to use an Android smartphone
- Dutch speaking and understanding

**Exclusion criteria for patient recruitment in the study.**

**Exclusion criteria:**

- Recent percutaneous coronary intervention or coronary artery bypass grafting procedure and still included in a cardiac rehabilitation program
- Orthopedic, neurologic, or any other pathologic condition that makes the patient physically unable to follow an app-based telerehabilitation program
- Planned interventional procedure or surgery in the next 4 months
- Pregnant females
- Present cardiovascular complaints
- Participation in other cardiac rehabilitation program trials, focusing on exercise outcome
- Any condition, which, in the opinion of the investigator, would make it unsafe or unsuitable for the patient to participate in this study or a life expectancy of less than 4 months based on investigators’ judgment

### Qualitative Analysis

**Figure 2:**
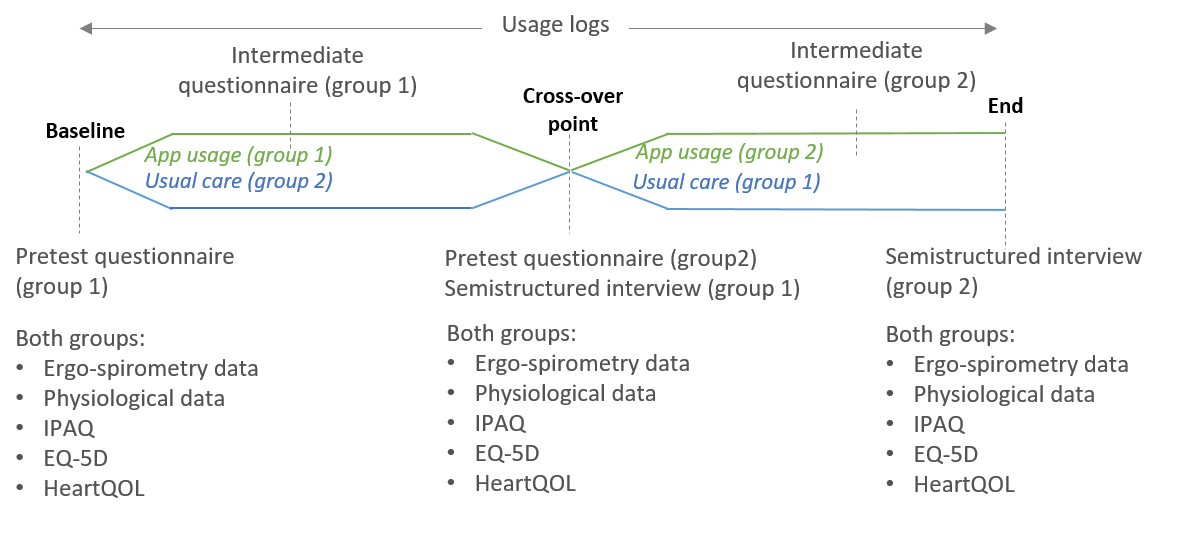
Qualitative and quantitative data collected across different time points of the study. IPAQ: International Physical Activity Questionnaire; EQ-5D: EuroQol 5-Dimensions questionnaire.

Qualitative data were acquired using pretest and intermediate questionnaires collected at baseline and end of 4 weeks in the app usage phase of the study (Figure 2). In addition, 2 HCI researchers conducted a final semistructured interview with patients at the end of their app usage phase. The questionnaires used are appended in Multimedia Appendix 2. All patients received an initial elicitation questionnaire to gather information on their familiarity and comfort with using smartphones and their current approach to log and monitor various aspects of their rehabilitation such as physical activities, medication, physiological parameters, and how they seek further information. During the course of their app use phase, at the end of 4 weeks, they were sent the intermediate questionnaire to gather information on how they perceive the influence of various modules of the app on their telerehabilitation progress, influence on their lifestyle, and motivation. These aspects were evaluated on a 5-point Likert scale. The values collected in the prestudy and intermediate questionnaires were used to probe and tailor questions about the app’s influence on their overall health, rehabilitation, and motivation during the final semistructured interview. The interview gave a more nuanced insight on patient perceptions and reasoning behind their perceptions, which cannot be gathered merely through questionnaires. The researchers conducting these interviews took notes, and audio recordings were made of all interviews. We then translated these interviews from Dutch to English and transcribed the responses. The findings were categorized into contexts such as overall experience with HeartHab, insights per module of the app, understandability of visual representations, perceptions on caregivers’ intervention, most helpful aspects of the app, features in the app that the patients felt were lacking or missing, and their motivation to continue using the app. We used a validated computer-assisted qualitative data analysis software (NVivo–developed by QSR International) [24,25] to do a context-wise sentiment analysis [26]. We then did a manual in-depth analysis of reasons associated with each positive or negative response.

### Quantitative Analysis

Descriptive analyses were performed on data collected using the app and through the various questionnaires (Figure 2). Data collected from the app includes usage logs (logging all interactions with the app including features that were accessed, frequency, the time spent on a certain feature, etc), activities registered by patients using the app, their medication compliance, and evolution of various physiological parameters. Data collected from the questionnaires included overall physical activity levels and quality of life indicators using International Physical Activity Questionnaire (IPAQ) [27], HeartQoL [28], and the 5-level EuroQol 5-Dimensions (EQ-5D) questionnaires [29]. Clinical parameters such as weight, blood pressure, heart rhythm, exercise capacity (maximal oxygen consumption [VO_2_ max]), glucose (glycated hemoglobin [HbA_1c_]), and lipid profile (low-density lipoprotein and high-density lipoprotein [HDL] cholesterol) were collected using blood tests and ergo-spirometry tests at the hospital and rehabilitation center. All measurements were procured at baseline, the crossover point (month 2), and the end (month 4). The gathered data were used to observe mean differences between various parameters during different stages of the study, compare assessments, and make preliminary estimates of the influence of the app on the different evaluation metrics (parameters listed in Multimedia Appendix 3). To gain a precise insight on the actual influence of the app, we used the overall app usage percentage of each patient as a weighting variable against which all parameters were weighted. The overall app use percentage was computed using the number of days of actual app use against the total days that a patient was in the app usage phase. We then performed a detailed weighted statistical analysis on the data according to the intention-to-treat principle by the assigned treatment group. Nonparametric alternatives (such as Wilcoxon 2-sample test) were used for parametric statistics (*t* tests) in case assumptions for the latter were violated.

#### Test for Significance

**Figure 3:**
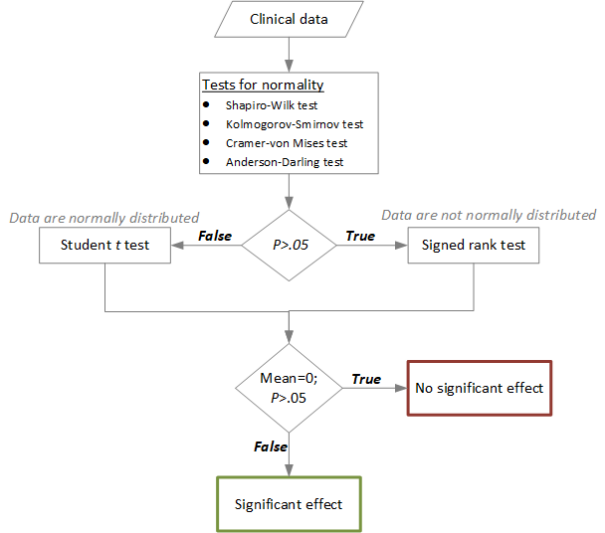
Flowchart showing the statistical analysis process to evaluate the effect of HeartHab on various health metrics.

For all the evaluations, the level of significance was 2-sided alpha=.05. First, we tested if the data were normally distributed using 4 tests for normality—Shapiro-Wilk test, Kolmogorov-Smirnov test, Cramer-von Mises test, and the Anderson-Darling test. When the data were normally distributed, we used Student *t* test, and when the data were not normally distributed, we used signed rank test to determine significant effects (Figure 3).

#### Test for Carryover Effects

**Figure 4:**
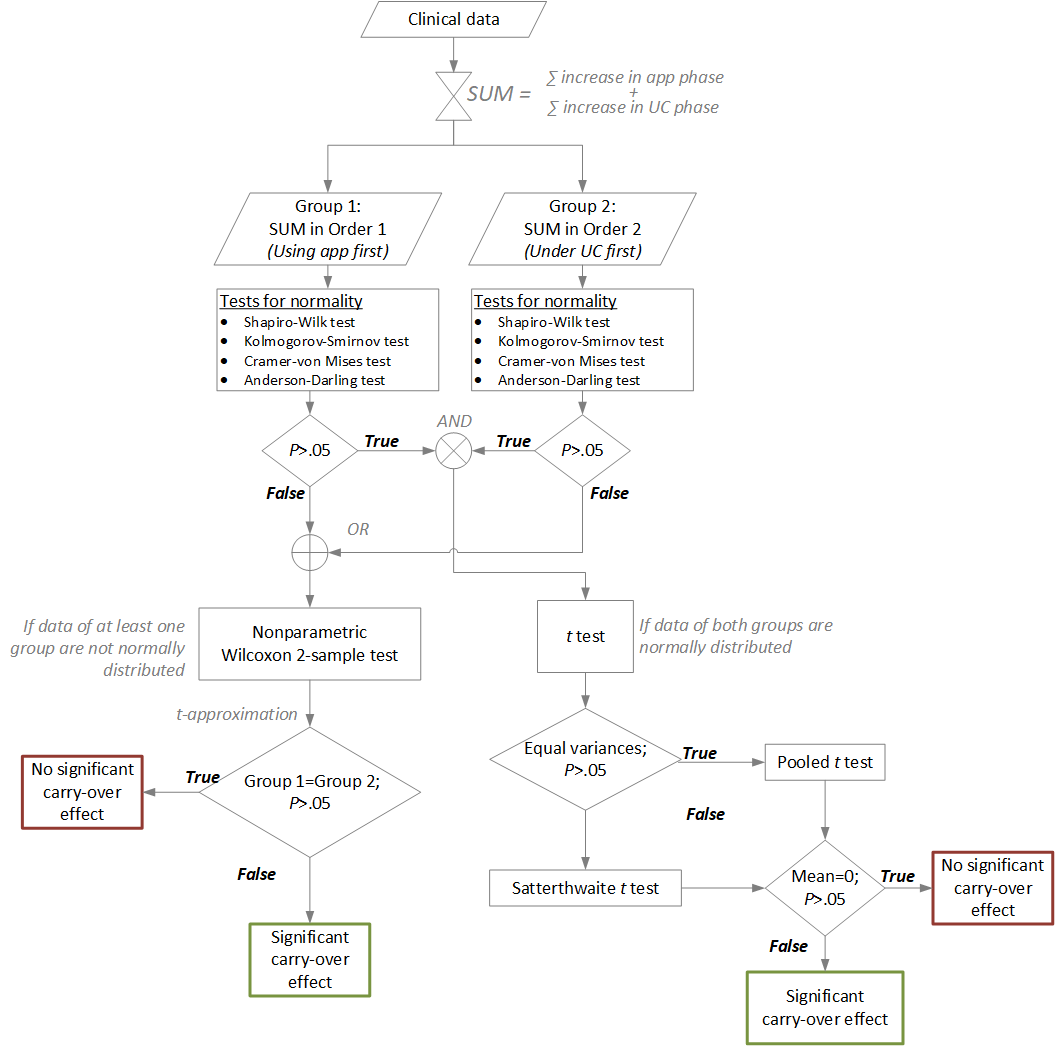
Flowchart showing the process of statistical analysis to identify significant carryover effects. UC: under control; SUM: summation.

Being a crossover study design, we also evaluated if there were any significant carryover effects. The primary objective to determine this was to see if any significance observed during the first phase with patients in group 1 (using the app in the first phase) was carried over when they switched to usual care in phase 2 as a means to get an indication on maintenance effects. The process followed to evaluate the carryover effect is detailed in the flowchart (Figure 4).

### Analysis of Subjective Measures

In addition, more information on physical activity and quality of life were procured through standardized questionnaires such as IPAQ, HeartQoL, and the 5-level EQ-5D. The methods followed to analyze these measures are detailed in the following subsections.

#### Physical Activity Data

The physical activity data collected using the IPAQ were translated into the volume of activity computed by weighting each type of activity by the energy requirements of that type of activity. The energy requirement is represented in terms of Metabolic Equivalents of Task (METs). This then yields a score in MET-minutes calculated by multiplying the METs of an activity by the duration for which it is performed and the resting metabolic rate as defined in IPAQ’s scoring protocol [30]. In HeartHab, for prescribing exercise targets and to follow progress made by patients, the METs per activity were procured from the compendium of physical activities [31]. Then alongside resting metabolism and duration of an activity, factors such as intensity at which the activity was performed and the patient’s weight were factored in, to get a more precise METs score per individual. The weekly METs scores achieved by each patient were compared against a personalized target range of METs that was prescribed by physiotherapists using the caregivers’ dashboard application. The dashboard application integrates the European Association of Preventive Cardiology–supported EXPERT tool for exercise recommendations [32]. The EXPERT tool facilitates physiotherapists and other clinicians to generate tailored exercise recommendations based on the patient’s individual pathology and risk factors.

#### Quality of Life Data

Quality-adjusted life years (QALYs) was used as a generic measure of effectiveness to assess change or evolution in the quality of life. Estimates of QALYs were derived from the EQ-5D questionnaire. The EQ-5D scores were converted to QALY indices based on the time trade-off technique and conversion weights determined for the Belgian population as per the EQ-5D evaluation protocol [33]. The EQ-5D also uses a visual analog scale (VAS) where patients can pick a score from 0 to 100 (0 being the *worst imaginable* health state and 100 being the *best imaginable* health state) based on their perception of their current health status. The mean VAS scores across different phases were also computed to see if there was a perceived change in the quality of life of patients before or after using the app. In addition, the scores reported in the HeartQoL questionnaire were used to determine the influence of their heart disease on different aspects of physical and emotional well-being. The change in QALY index, VAS, and heart health score across different phases of the study is used to evaluate the impact of the app on the quality of life of patients.

## Results

In this section, we present qualitative, quantitative, and comparative results for the 4 factors of CR identified in the goal of this study—F1) patients’ perspectives, experience, and impact on motivation; F2) impact on physical activity; F3) impact on quality of life; and F4) impact on risk factors. Detailed statistical outcomes with respect to the impact of use of HeartHab on various physiological parameters, crossover impact, and correlations are presented in Multimedia Appendix 3.

### Data Exclusion

**Figure 5:**
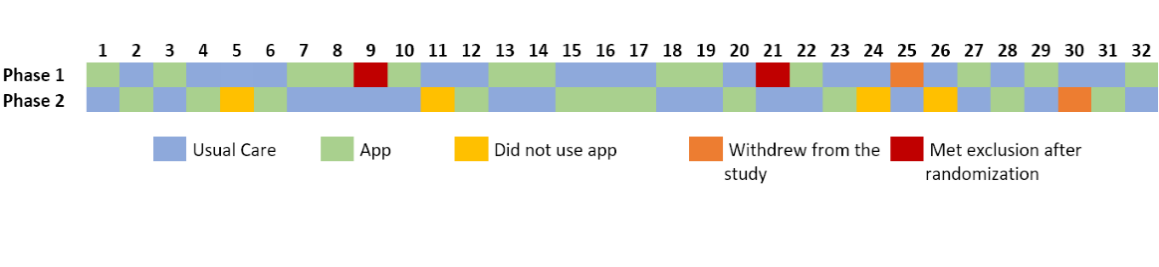
Representation of all patients recruited in the study, their randomization sequence, and app usage.

Overall, 2 patients were excluded after randomization when they developed other comorbidities such as back problems or encountered another cardiac incident. Therefore, they did not complete the study. In addition, 2 others were excluded as they chose to withdraw from the study during or before startup. The baseline patient information of the remaining 28 patients is presented in Table 1. Given the design of our study, the decision to exclude the data of these patients was made after discussions with a statistician. Patients that had missing data in the IPAQ questionnaire [27] or had overreported their physical activity effort according to IPAQ’s scoring protocol [30] were excluded in the analysis of overall physical activity (F2). Patients with missing data in either the EQ-5D [29] or HeartQoL [28] questionnaires were excluded in the assessment of *“Quality of Life”* (F3). Figure 5 illustrates all the patients who were recruited with their assigned randomization, usage levels, and exclusion criteria.

**Table 1:** Presentation of baseline patient features, cardiac pathology, and medication information (n=28).

General patient features		Baseline values
Age (years), mean (SD)		60.9 (8.2)
Gender, n		24 males and 4 females
Body mass index (kg m s^−1^), mean (SD)		28.7 (5.2)
**Cardiac pathology, n**		
	Coronary artery disease, percutaneous coronary intervention, coronary artery bypass grafting, and endoscopic atraumatic coronary artery bypass	28
	Cardiac resynchronization therapy, pacemaker, and implantable cardioverter defibrillator	1
	Pulmonary arterial hypertension	1
**Medication, n**		
	Calcium antagonists	2
	Beta-blockers	17
	Angiotensin-converting enzyme inhibitors	19
	Statins	25
	Antiplatelets	28

### F1) Patients’ Perspectives, Experience, and Impact on Motivation

**Figure 6:**
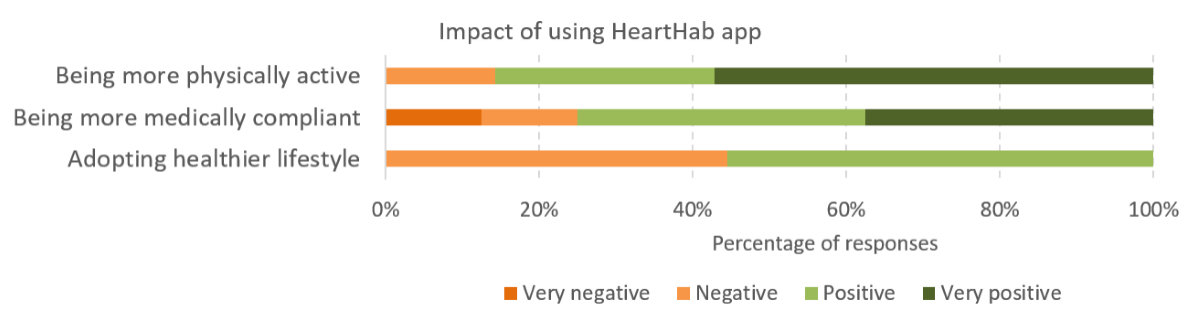
Results of sentiment analysis using NVivo on increasing physical activity levels, promoting medication adherence and adopting healthier lifestyles after using the HeartHab app.

Most patients were positive on the impact of using HeartHab on being more physically active and more medically compliant (Figure 6). Regarding how the app promoted being more physically active, P20 remarked:

I have been a bit more active than usual, and it also helped a lot that I could follow up all my activities. It pushed me to do a bit more than I usually tend to do.

Another patient stated the following:

My efforts have increased. The flag [referring to the persuasive visualization of exercise target in the app’s interface]was motivating. It [pointing to the goals and progress on the app] motivates you to exercise more.

P18

There were mixed responses on adopting a healthier lifestyle. Although some patients perceived the influence on physical activity and medication compliance as aspects of healthier lifestyle, some patients opined that they had already tried to adopt a healthier lifestyle as their diagnosis and the app did not change much:

Actually, the application should be used immediately in rehabilitation. Then the app is probably more useful than with me, but it's all right.

P26

On the other hand, P20 said:

I lost 7 kilograms. It [pointing to the phone/app] was one of the factors which pushed me towards a healthier life. You are aware of the fact that you are in a study and the fact that the app reminds you and nudges you at the correct time helps a ton.

**Figure 7:**
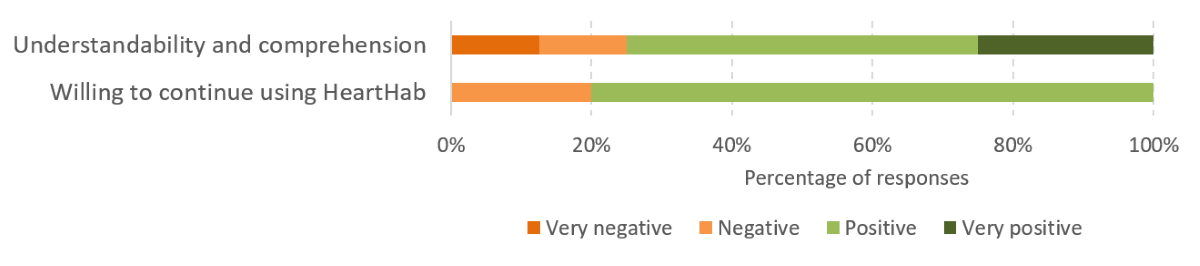
Results of sentiment analysis using NVIVO on patients' motivation to continue using HeartHab and the impact of the app on improving their overall understandability and comprehension.

Most patients were positive about the support provided by the app in their rehabilitation, and 80% of the patients were willing to continue using the app to support long-term self-management (Figure 7). For example, when asked about their motivation to continue using HeartHab, P11 said:

Too bad it is being taken away[when the app was uninstalled from his phone at the end of the study].It was a luxury to have it. I had my entire heart history...good background information and it can also be useful for the cardiologist.

Another patient said:

Yes of course. I don’t have to hesitate about that [referring to willingness to continue using HeartHab] for a second. There exist many applications [referring to other generic health apps] that are less useful than this one.

P7

For patients who did not want to continue using the app, it was mainly because they believed that they already lead an active and healthy lifestyle and did not necessarily need an app. Other patients were not looking forward to continue using the app because of their discomfort in using smartphones. P2 also remarked:

My wife helps me now. The app is more interesting for singles [possibly referring to people that do not have an active informal caregiver] and poorly motivated people.

Another patient said the following:

I am already quite active. I just have to watch my cholesterol but this [referring to the app] did not help me a lot further.

P27

### F2) Impact on Physical Activity Levels

**Figure 8:**
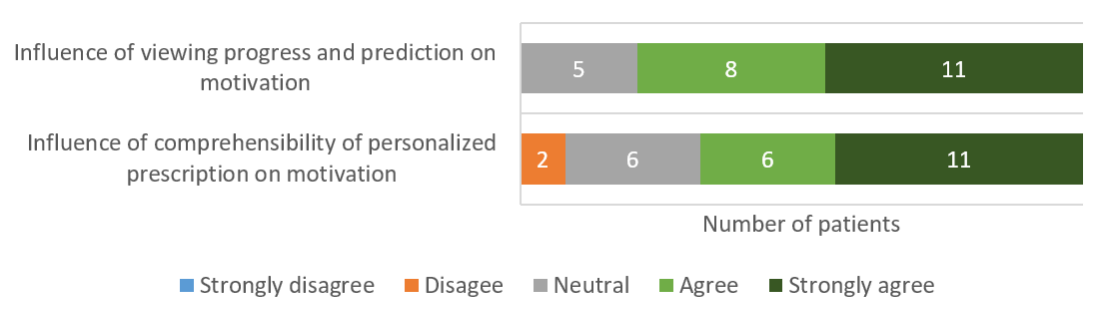
Patients' perceptions on the impact of HeartHab on motivation to achieve physical activity targets as collected in the intermediate questionnaire.

**Figure 9:**
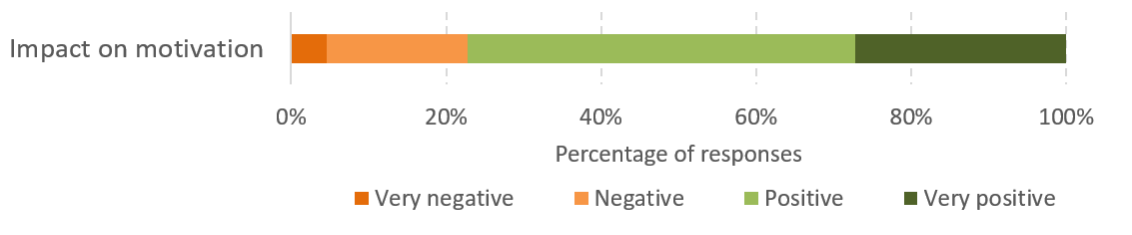
Sentiment analysis using NVivo on the impact of various aspects of HeartHab on motivation to be more physically active.

The progress and prediction visualizations facilitate in nudging patients to reach their targets. In both the intermediate questionnaire (collected after 4 weeks in the app use phase; Figure 8) and the final semistructured interview, most patients responded positively about both these elements (Figure 9). Therefore, the persuasive design approach applied in the app [21] certainly had an influence on the motivation of patients:

I try to exercise more. I would aim to get to the flag [referring to the visualization of targets in the app]. It was not always successful because of lack of time. But it certainly motivated me.

P18

I really liked the fact that I could explore the effect of different activities on the goals! So I chose my activity sometimes based on the effect it would have on my progress towards the goals.

P10

Here, the patient refers to the feature in the app that predicts the effect of an activity in terms of METs, thus facilitating the selection of activities that contribute enough to gradually reach the personalized target.

Even patients who were already active and did not necessarily increase their physical activity found the app motivating and felt reassured when they saw their progress:

I was already active. So, I did not do more. But it was nice to see progress... liked the motivational messages the app would give after entering a new activity such as “keep going”, “good job”etc...

P12

**Figure 10:**
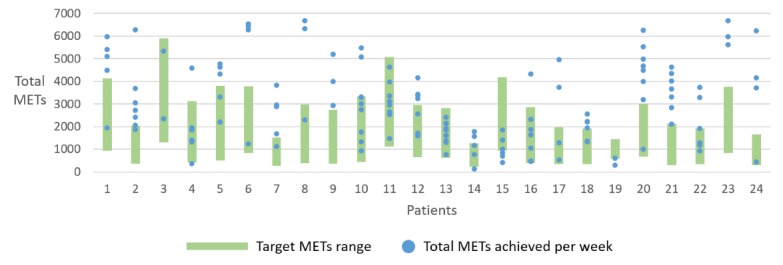
Chart showing weekly physical activity targets and totals METs achieved each week by patients that used the physical activity module of the HeartHab app. The chart depicts only complete data for weeks when a patient registered activities. MET: Metabolic Equivalents of Task.

These subjective outcomes were also reflected in the target achievement rates that were measured using the app. Overall, 84% (21/25) of patients either reached or exceeded their maximal target on average (Figure 10). The targets were tailored individually based on their exercise capacity as measured in the ergo-spirometry test. For example, P1 was prescribed to undertake exercise training for 7 times a week for a duration of 20 to 60 min per session based on his exercise capacity, which yielded a minimal weekly target of 921 METs (ie, 20 min/session and 7 sessions) and a maximal target of 4145 METs (ie, 60 min/session and 7 sessions). On the other hand, P7 who had a much lower exercise capacity was prescribed to undertake exercise training for 3 to 5 times a week for a duration of 20 to 45 min per session. This resulted in a minimal target of 269 METs (ie, 20 min/session and 3 sessions) and a maximal target of 1512 METs (ie, 45 min/session and 5 sessions). This personalized tailoring of targets motivated better target achievements and added to the credibility of the app as reflected by the following patient perceptions:

...it [the targets] seemed correct and trustworthy for me, realistic; otherwise I would not have looked at the activity goals.

P10

I assume that it is credible because it is prescribed by the physiotherapist. That motivates me.

P18

The goals were achievable and made for continuous use

P2

In the study, the duration of app usage varied between 7 to 10 weeks for all patients. The representation of weekly METs achievement in Figure 10 only shows values that were registered by patients in the app. There were weeks where some patients did not register all activities, and the incomplete data are not visualized in the chart.

**Figure 11:**
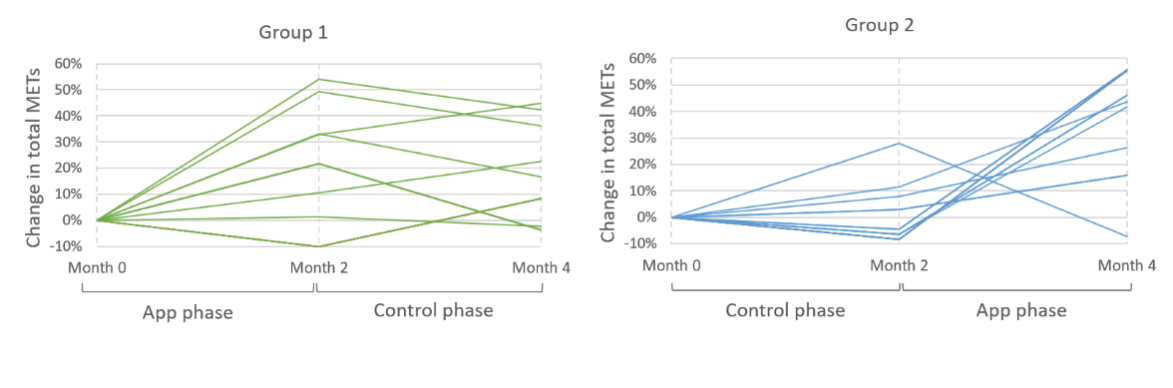
Graphs showing increase or decrease in total Metabolic Equivalents Of Task across different phases of the study for both groups of patients as collected from the International Physical Activity Questionnaire. MET: Metabolic Equivalents of Task.

As inferred from the graphs (Figure 11) for both groups of patients, there was a clear increase in total METs during the app phase. For some patients in group 1 (ie, patients who used the app in the first phase), we can also see the increasing trend continue onto the control phase. The increase we observe with patients in group 2 upon starting the app also suggests the positive impact of the app on increasing physical activity levels. For 1 patient in group 2, there is a substantial increase in the control phase and a steep drop in the app phase. The increase in control phase was due to a regional sporting event in which the patient participated. Therefore, the trend seen in this graph does not reflect on the patients’ normal activity behavior.

Although the data of all patients are not visualized in the graphs because of incomplete data in the IPAQ questionnaires, the statistical evaluation of exercise capacity in terms of VO_2_ max obtained from the ergo-spirometry tests across the phases showed a positive significant carryover effect (*P*=.008; Multimedia Appendix 3). This indicates the maintenance of acquired exercise capacity even upon completion of intervention (use of app), which was one of the key goals of this study.

**Figure 12:**
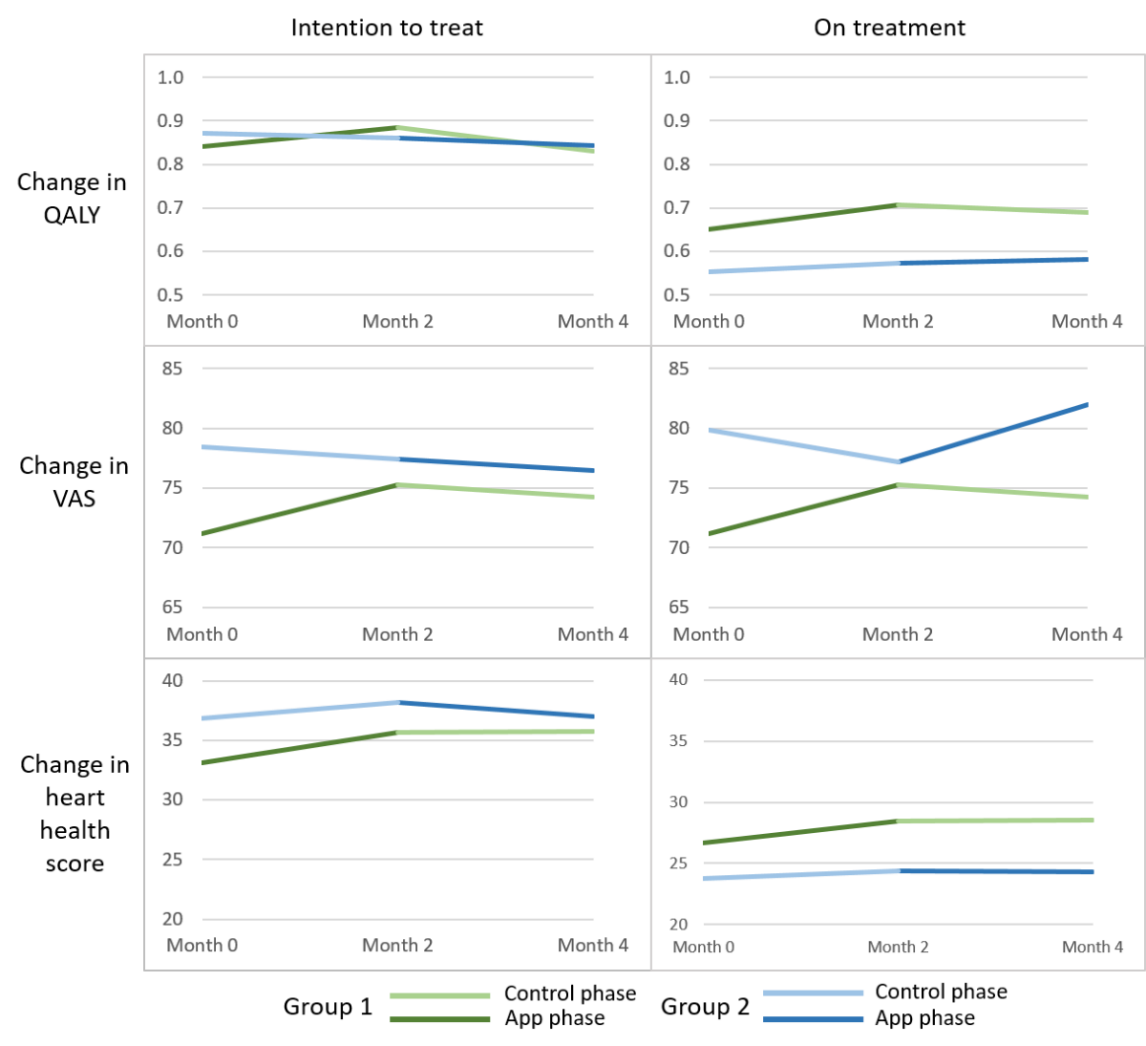
Changes in various assessments of quality of life across different phases of the study. QALY: quality-adjusted life years; VAS: visual analog scale.

### F3) Impact on Quality of Life

Figure 12 depicts the quality of life data obtained from EQ-5D and HeartQoL questionnaires as assessed in 2 arms—intention-to-treat and on-treatment. In the intention-to-treat approach, the data of all patients that completed the study were included. For the on-treatment approach, the data of patients were excluded if there were any incomplete data in any of the phases of the study or if the patient developed a new symptom such as back pain, which was not directly caused by the app.

On the basis of the EQ-5D questionnaire, there was a mean increase of 0.06 QALY and a mean decrease of 7.14% in anxiety during the app usage phase for both groups of patients using the intention-to-treat analysis. From VAS in Figure 12, we can observe a clear trend in the perceived increase of health during the app phase by both groups of patients, which was lacking in the control phase. We could also see a marginal increase in heart health scores obtained from the HeartQoL questionnaire.

### F4) Impact on Risk Factors

On the basis of the blood tests taken at baseline, crossover point (end of month 2), and final (end of month 4) appointments, we found a significant increase in HDL, which is also nicknamed *“the good cholesterol”* with *P*=.048 (*d*=0.78; 95% CI 0.02-1.55). There was a mean increase of 0.61 mg/dL in HDL cholesterol in the app usage phase as compared with a mean decrease of 2.28 mg/dL during the control period, demonstrating a clear impact of app use on this parameter. In addition, we found a significant decrease in HbA_1c_ with *P*=.01 (*d*=1.03; 95% CI 0.24-1.82). HbA_1c_ gives the glucose concentration in blood, which gives an indication of average blood sugar levels over a period. Although there was a mean decrease of 0.3 mg/dL in HbA_1c_ in the control period, there was a mean decrease of 1.5 mg/dL during the app usage period. Therefore, a significant decrease in this parameter in the app phase indicates a significant decrease in risk of diabetes-related complications. The increase in overall physical activity is reflected in the positive significant effects on these parameters as physical activity does have an influence on both HbA_1c_ and HDL. With respect to other risk factors such as weight (*d*=0.4076) and pulse (*d*=0.4518), we observed a positive trend with a mean decrease in weight of 0.02 kg and a mean decrease in pulse of 3 bpm, although the results were not statistically significant.

## Discussion

### Reflections on Outcomes of the Study

The qualitative findings put forward a nuanced interpretation of how motivated patients feel across different phases of the study. The perceptions of patients may not always map to the quantitative results, but gathering those findings is equally important. For example, a patient who did not like to sport or engage in rigorous physical activity mentioned the following:

...I either walked or biked to the supermarket instead of taking my car so I can register it on the app.

P10

Such small changes in health behavior as gathered from qualitative evaluations cannot be observed purely from quantitative measures and are, nonetheless, potentially beneficial for this patient population. These observations are also suggestive of the impact of the persuasive elements of the HeartHab app on the motivation of patients.

The findings presented in this paper establish the positive effect of the app of study goals F1 (patients’ perspectives, experience, and impact on motivation) and F2 (impact on physical activity). The impact on physical activity is also reflected in the significant effects on other parameters such as glucose and cholesterol. In addition, the carryover effects that we observed in this study are clinically important because one of the main challenges in telerehabilitation is the high rate of dropout and nonadherence after the intervention concludes [9]. Therefore, it is important for telerehabilitation to support and gradually train patients toward self-management. In this study, we found significant carryover effects on weight, HDL, and VO_2_ max, indicating the sustenance of motivation and intervention effects at the end of the intervention. The willingness of patients to continue using the app at the end of the study is a promising indication toward minimizing attrition and dropouts. Moreover, patients’ perceptions on the influence of viewing progress and prediction on their motivation and target achievement indicates how the app could support better self-awareness and promote self-management. Furthermore, we observed clinically significant effects on cholesterol and glucose. The effect on glucose concentration (*d*=1.03) is higher as compared with cholesterol (*d*=0.78) as patients have a better control over their insulin intake and sugar consumption. Effects on other parameters are not clinically significant and could be attributed to the relatively short duration of this study. Finally, the reduction in anxiety and perceived increase in quality of life emphasize on the positive effect of the app on goals F3 (impact on quality of life) and F4 (impact on risk factors) of the study.

### Implications for Future Work

We acknowledge that the reported results are representative of only a small sample studied at 1 rehabilitation setting where all patients underwent the same supervised rehabilitation program and received similar advice on maintenance and follow-up with CR. It might, therefore, be interesting to evaluate the impact of the app on patients across different settings and in different rehabilitation phases. The subjective perceptions could also vary when studied under these different settings. However, by detailing our approach and findings, we hope to enable researchers to make an informed assessment of the adoption of similar methods within their practice or research contexts. Adding intelligibility to enable patients to better comprehend their rehabilitation progress and tailoring the intervention to suit individual needs seem highly useful in persuading patients. Future research could assess these factors in a longer duration to also study how patients’ engagement varies across different phases of intervention and after the conclusion of the intervention. We believe that such integration of persuasive techniques in app development and evaluation using a mixed-methods approach can add to the existing knowledge base of technology-supported cardiac telerehabilitation and pave way for more such multidisciplinary research in this domain.

### Conclusions

We conducted a multidisciplinary crossover study with 32 cardiac patients, and 28 patients successfully completed the study using the HeartHab app. We found positive significant effects of the app-based telerehabilitation approach on HbA_1c_ (*P*=.01; *d*=1.03; 95% CI 0.24-1.82) and HDL cholesterol (*P*=.04; *d*=0.78; 95% CI 0.02-1.55). We also found positive significant carryover effects on VO_2_ max, HDL, and weight. The persuasive techniques applied in HeartHab yielded a positive outcome on patient motivation and facilitated them in achieving overall physical activity targets. The insights gathered from the study could help address some of the challenges faced by current systems targeting cardiac telerehabilitation and secondary prevention. A longer evaluation with a larger group of patients can potentially lead to other significant health outcomes and address remaining challenges that are currently hampering the effective widespread implementation of cardiac telerehabilitation.

## Appendix

Multimedia Appendix 1 Video walkthrough of the HeartHab mobile application.

Multimedia Appendix 2 Human-computer interaction questionnaires that were used for collecting qualitative data across different phases of the study.

Multimedia Appendix 3 Statistical data analysis for effects of HeartHab on various parameters and carryover effects.

## Abbreviations

CAD: coronary artery disease
CR: cardiac rehabilitation
EQ-5D: EuroQol 5-Dimensions
HbA_1c_: glycated hemoglobin
HCI: human-computer interaction
HDL: high-density lipoprotein
IPAQ: International Physical Activity Questionnaire
MET: Metabolic Equivalents of Task
QALY: quality-adjusted life years
VAS: visual analog scale
VO_2_ max: maximal oxygen consumption
