# Supplementary material for: Evaluating the Impact of the HeartHab App on Motivation, Physical Activity, Quality of Life, and Risk Factors of Coronary Artery Disease Patients: Multidisciplinary Crossover Study
Source: JMIR Mhealth Uhealth. 2019 Apr 4;7(4):e10874. doi: 10.2196/10874 (PMC6470465; doi:10.2196/10874)
Supplement: Multimedia Appendix 2 [file mhealth_v7i4e10874_app2.pdf]

HeartHab Questionnaires

Pre-test questionnaire

Demographics

1. Date of birth: …
2. Gender: M / F
3. What is your highest educational qualification? ……………………………………………………………
4. What is your occupation? ………………………………………………………………………………………….

Experience with technology

5. How frequently do you use the following devices?

	I do not know	Never	Few times a month	Few times a week	Daily
Computer / laptop					
Tablet					
Smartphone					
Generic mobile phone					
Smartwatch					

6. For what purpose do you use the following devices?

	Personal use	For work	I do not use it
Computer / laptop			
Tablet			
Smartphone			
Generic mobile phone			
Smartwatch			

7. For which of the following do you use the following devices?

	Communication (calls, SMS, e-mail, chat, etc.)	Searching information	Playing games	Listening to music	To view / shoot pictures / videos	Social networking (eg. Twitter, Facebook)	I do not use it
Computer / laptop							
Tablet							
Smartphone							
Generic mobile phone							
Smartwatch							

8. How much do you like using technology and applications?

○	○	○	○	○
Do not like at all	Do not like	Neutral	Like	Like a lot

9. Do you have experience with using other mobile health apps?
   - o Yes
   - o No

   If yes, which ones?
   ……

   If yes, how much do you like using these apps?

○	○	○	○	○
Do not like at all	Do not like	Neutral	Like	Like a lot

10. How often do you use the following health measurement devices?

	I do not know	Never	Few times a month	Few times a week	Daily
Weighing scale					
Heart rate monitor					
Blood pressure monitor					
Pedometer or activity tracker (e.g. Fitbit)					
Others…					

Medication

11. At this moment, how do you organize the different medication that you have to take?
    - ☐ Pillboxes with time of the day (e.g. morning, noon, evening)
    - ☐ Notes (e.g. Post-it notes)
    - ☐ Others: ………………………………………………….
12. Do you forget to take your medication?
    - o Yes
    - o No

    If yes, how frequently do you forget?

    ………

    ………

Physical activity

13. Do you keep track of your physical activities at the moment?
    - o Yes
    - o No

    If yes, how do you keep track of it?

    ………

    ………

14. How much do you like to engage in sports/physical exercise?

○——○——○——○——○

Do not like at all	Do not like	Neutral	Like	Like a lot

15. What is your perception of physical exercise? *(you may select multiple options)*
    - ☐ Recreation
    - ☐ Work out
    - ☐ Enjoying nature
    - ☐ Getting some fresh air
    - ☐ Commuting/Travel
    - ☐ Compulsory exercise for rehabilitation
    - ☐ Others: ………………………………………………………………………………………………..

Medical parameters

16. Do you track your medical parameters at the moment (e.g. Blood pressure, weight etc.)?
    - o Yes
    - o No

    If yes, how do you keep track of it?

    ……

    ……

Information

17. Through which of the following sources do you try to find out more about your health condition? *(you may select multiple options)*
    - ☐ Online videos
    - ☐ Books, magazines, brochures
    - ☐ Online search
    - ☐ Talking to my doctor / dietician / physiotherapist
    - ☐ Others: ……………………………………………………………………………………………………….

Intermediate Questionnaire

1. How do you feel at the moment?

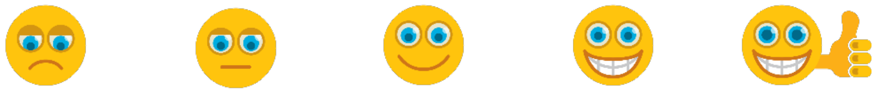

1 2 3 4 5

2. Has the HeartHab application changed the way you perform your physical activities?
   - o Yes
   - o No

   If yes, in which manner has the HeartHab application changed the way you perform physical activities?

   ……

3. Has the HeartHab application changed the way you take your medication?
   - o Yes
   - o No

   If yes, in which manner has the HeartHab application changed the way you take medication?

   ……

4. Were you afraid in the past month because of your health condition?
   a. Yes
   b. No

   If yes, can you describe when you were afraid? (e.g. during a physical activity, because you forgot your medication, you had to travel somewhere, you had a medical examination,...)

   ……
   ……

Rate the following statements:

5. Being remotely monitored by the caregivers helped reduce my fear.

Strongly disagree	Disagree	Neutral	Agree	Strongly agree
o	o	o	o	o

6. Being remotely monitored by the caregivers motivated me to be more physically active or more medically compliant.

Strongly disagree | Disagree | Neutral | Agree | Strongly agree

7. Rate the following statements about the use of the HeartHab application:

	**Strongly disagree**	**Disagree**	**Neutral**	**Agree**	**Strongly agree**
The HeartHab application is easy to use.					
The guidance of the HeartHab application feels reliable.					
The information presented in the HeartHab application is easy to understand.					
The HeartHab application motivates me to be more physically active.					

8. Evaluate the following statements about specific components of the HeartHab application *(Note that the images contain fictitious data)*:

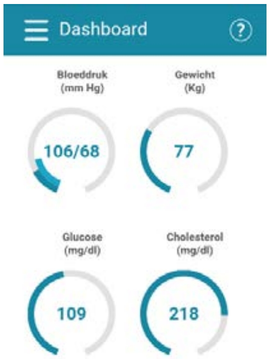

a) The feedback shown on the dashboard page motivates me to strive for a healthier lifestyle.

Strongly disagree | Disagree | Neutral | Agree | Strongly agree

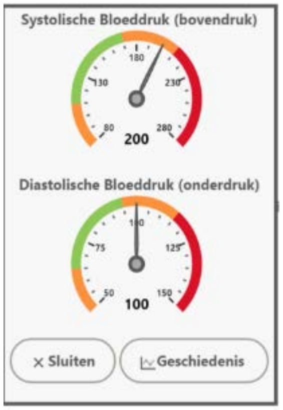

b) The colors in the dashboard details motivates me to adopt a healthy lifestyle.

Strongly disagree — Disagree — Neutral — Agree — Strongly agree

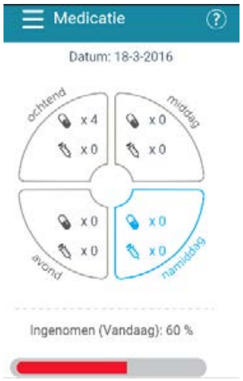

c) The bar showing my medication adherence motivates me to be more compliant.

Strongly disagree — Disagree — Neutral — Agree — Strongly agree

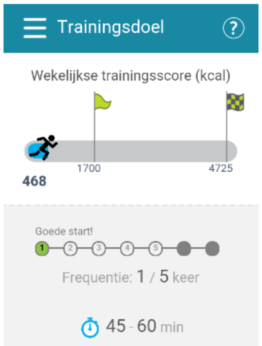

d) Being able to see my progress to what extent I have achieved my goals motivates me to be more physically active.

Strongly disagree	Disagree	Neutral	Agree	Strongly agree

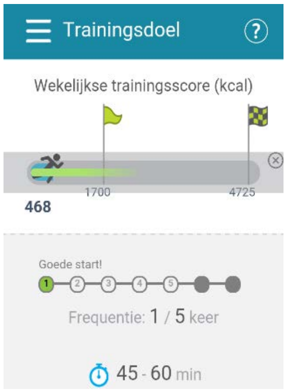

e) Viewing the estimated prediction is very useful to know if my current efforts are sufficient to achieve my goals.

Strongly disagree	Disagree	Neutral	Agree	Strongly agree

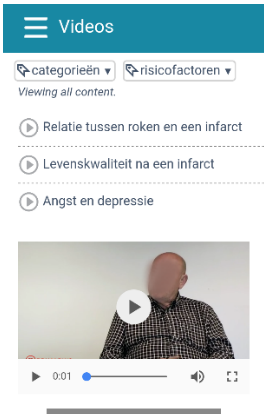

f) Viewing the videos is very useful to learn how to adapt my lifestyle.

Strongly disagree	Disagree	Neutral	Agree	Strongly agree
○	○	○	○	○

g) The videos help me to better understand various aspects concerning my health condition.

Strongly disagree	Disagree	Neutral	Agree	Strongly agree
○	○	○	○	○

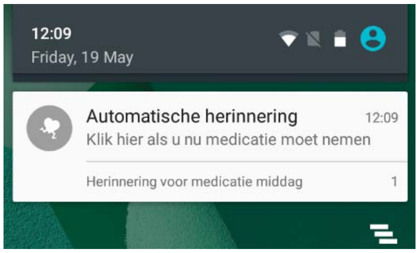

h) The reminders and notifications were very useful.

Strongly disagree	Disagree	Neutral	Agree	Strongly agree
○	○	○	○	○

i) The reminders and notifications prompted me to take necessary actions.

Strongly disagree	Disagree	Neutral	Agree	Strongly agree
○	○	○	○	○

9. Did you experience any problems with the HeartHab application, the smartphone or possibly additional measuring devices during the past month?
………
………
………
………
……………

10. Would you like to share any other experience regarding the use of the HeartHab application, or give some suggestions?
………
………
………
………
……………

Questions for semi-structured interview

1. How was your overall experience with using the HeartHab application?
   a. Did using HeartHab encourage you to adopt a healthier lifestyle?
   b. Did using HeartHab encourage you to be more physically active? Did your efforts increase, decrease or remain the same?
   c. Did using HeartHab encourage you to follow your medication? Did your compliance increase, decrease or remain the same?
   d. Were the coaching videos informative? Did you learn something from those videos that made you change a specific aspect of your lifestyle? (e.g.- Knowing more does not change my lifestyle, Its very useful for me to learn useful exercise/diet tips, Helps me lowering my symptoms, Interesting but not applicable to me etc..)
2. Were the visual representations understandable? Did you struggle with comprehending certain aspects or elements?
3. Module specific questions: *check their responses for each module in the intermediate questionnaire and add-to/adapt the below questions accordingly.*

   Main aspects to be covered for each module- Understandability, perceived usefulness and impact on motivation (we could again use scores of 10 to make it easier)

   a. Dashboard: Was the overview shown in dashboard useful? Were the color-codes helpful in giving you insights on your parameters? Did it urge to you take special steps to control the parameters that were not optimal? (e.g. check medication intake/ diet if blood pressure/glucose was high).
   b. Medication: Was the overview in quadrants and prescriptions useful? Did having the compliance bar have an influence on promoting adherence? Were the reminders helpful? Did reminders have a specific influence on promoting adherence?
   c. Activities: Were the weekly goals in terms of total score, sessions and duration understandable? Did seeing the progress have a specific impact on motivating you to do more physical exercise?
   d. Coaching videos: Was the content easy to look for? Would it be better if they were filtered automatically to specific needs? Would you like to be notified by specific videos based on your parameters/compliance?
4. Caregiver intervention:
   a. Knowing that you were being monitored helped reduce fear and anxiety?

   b. Knowing that you were being monitored help you pursue your activity targets/ be more compliant?
   c. The credibility associated with the targets and parameters made you trust the system better/ had an influence on your level of motivation?
5. If the patient logged any symptoms; would they find it useful if that was being followed up actively?

   For the subsequent questions, we will have to check what the patients have listed in the observation sheet that was handed to them and try to go through it together with them:

6. What aspects/elements did you find particularly helpful/useful?
7. Any component they missed or might find useful for the future?
8. Would you be interested to continue using such a system in the future?
9. Points of improvement for the app?
10. Other comments/suggestions
