# Supplementary material for: Evaluating the Impact of the HeartHab App on Motivation, Physical Activity, Quality of Life, and Risk Factors of Coronary Artery Disease Patients: Multidisciplinary Crossover Study
Source: JMIR Mhealth Uhealth. 2019 Apr 4;7(4):e10874. doi: 10.2196/10874 (PMC6470465; doi:10.2196/10874)
Supplement: Multimedia Appendix 3 [file mhealth_v7i4e10874_app3.pdf]

Summary of statistical outcomes of the HeartHab study

Table 1. Table showing weighted effect of app on various parameters including p-values, effect sizes and confidence interval.

Parameter	Test for Normality	Mean	Student's t-test			Signed Rank Test			Conclusion
			p-value	Effect size (*d*)	95% C.I	p-value	Effect size (*d*)	95% C.I	
Weight	Normal	0.27	0.29	0.41	[-0.3408, 1.1561]				No significant effect
Pulse	Normal	-1.90	0.24	0.45	[-0.2984, 1.202]				No significant effect
LDL	Normal	-1.63	0.45	0.29	[-0.456, 1.0333				No significant effect
**HDL**	Not Normal	2.22				0.048	0.78	[0.0152, 1.5527]	Significant effect (neg)
Total Cholesterol	Normal	-1.36	0.59	0.21	[-0.5343, 0.9513]				No significant effect
**HbA1c**	Not Normal	-0.07				0.01	1.03	[0.2396, 1.816]	Significant effect
AE	Not Normal	0.89				0.59	0.21	[-0.5374, 0.9481]	No significant effect
AT	Normal	0.54	0.81	0.09	[-0.6494, 0.833]				No significant effect
VO_2max	Normal	-0.03	0.22	0.48	[-0.2719, 1.2309]				No significant effect
QoL	Not Normal	0.78				0.33	0.38	[-0.3685, 1.1264]	No significant effect
QALY	Not Normal	0.02				0.37	0.35	[-0.3981, 1.0947]	No significant effect
METs	Not Normal	766.72				0.49	0.27	[-0.4778, 1.0103]	No significant effect

Hypothesis: H0: mean = 0 (no effect); H1: mean is different from 0 (effect)

The effect size (d) is calculated using standardized mean difference method.

Table 2. Carry over effects of using HeartHab observed at the end of the cross-over study on various psysiological parameteres.

Parameter	Test normality			Test normality (Log)			Test for equality of variances		Test for equality of variances (Log)		Parametric test		Parametric test (Log)		Non-parametric test	Conclusion
	Seq. 1	Seq. 2	Conclusion	sequence 1	sequence 2	Conclusion	Equality of Variances	Conclusion	Equality of variances	Conclusion	Pooled	Satterthwaite	Pooled	Satterthwaite		
**Weight**	Normal	Normal	Normal				0.70	Equal			0.006					Carry-over effect
Pulse	Normal	Normal	Normal				0.19	Equal			0.128					No Carry-over effect
LDL	Normal	Not Normal	Not Normal												0.087	No Carry-over effect
**HDL**	Normal	Normal	Normal				0.03	Not Equal				0.721				Carry-over effect
Total Cholesterol	Normal	Normal	Normal				0.62	Equal			0.189					No Carry-over effect
HbA1c	Not Normal	Not Normal	Not Normal	Not Normal	Not Normal	Not Normal									0.613	No Carry-over effect
AE	Not Normal	Normal	Not Normal	Normal	Normal	Normal			0.892	Equal			0.321			No Carry-over effect
AT	Normal	Normal	Normal				0.87	Equal			0.183					No Carry-over effect
**VO_2max**	Normal	Normal	Normal				0.97	Equal			0.008					Carry-over effect
QoL	Normal	Normal	Normal				0.17	Equal			0.127					No Carry-over effect
QALY	Not Normal	Not Normal	Not Normal												0.731	No Carry-over effect
METs	Normal	Normal	Normal				0.11	Equal			0.82					No Carry-over effect

Hypothesis for test of equality of variances: H0: variances are equal; H1: variances are unequal.

Hypothesis for parametric tests: H0: mean sequence 1 = mean sequence 2; H1: mean sequence 1 is different from mean sequence 2.
